# Methylamine Activates Glucose Uptake in Human Adipocytes Without Overpassing Action of Insulin or Stimulating its Secretion in Pancreatic Islets

**DOI:** 10.3390/medicines6030089

**Published:** 2019-08-12

**Authors:** Christian Carpéné, Pascale Mauriège, Nathalie Boulet, Simon Biron, Jean-Louis Grolleau, Maria José Garcia-Barrado, Mari Carmen Iglesias-Osma

**Affiliations:** 1 Institute of Metabolic and Cardiovascular Diseases, INSERM, UMR1048, Team 1, 31432 Toulouse, France; 2 I2MC, University of Toulouse, UMR1048, Paul Sabatier University, 31432 Toulouse, France; 3 Department of Kinesiology, Faculty of Medicine, Laval University, Québec, QC G1V0A6, Canada; 4 Department of Surgery, Faculty of Medicine, Laval University, Québec, QC G1V0A6, Canada; 5 Department of Plastic Surgery, CHU Rangueil, 31059 Toulouse, France; 6 Laboratory of Neuroendocrinology, Institute of Neurosciences of Castilla y León (INCyL), University of Salamanca, 37007 Salamanca, Spain; 7 Laboratory of Neuroendocrinology and Obesity, Institute of Biomedical Research of Salamanca (IBSAL), University of Salamanca, 37007 Salamanca, Spain; 8 Department of Physiology and Pharmacology, Faculty of Medicine, University of Salamanca, 37007 Salamanca, Spain

**Keywords:** adipose tissue, obesity, amine oxidase, glucose transport, insulin sensitivity, semicarbazide

## Abstract

**Background:** Methylamine, a natural soluble amine present in foods, is known to be a substrate of primary amine oxidase (PrAO) widely expressed in animal tissues. Methylamine has been reported to activate glucose transport in fat cells and to facilitate glucose disposal in rabbits but the interests and limits of such insulin-mimicking actions have not been further explored. This work aimed to perform a preclinical study of the inter-individual variations of these biological properties to study the putative link between PrAO activity and insulin resistance. **Methods:** Methylamine was tested on human adipocyte preparations and in rabbit pancreatic islets to determine its influence on glucose uptake and insulin release, respectively. PrAO activity and related responses were determined in adipose tissues obtained from two cohorts of non-obese and obese women. **Results:** Adipose tissue PrAO activity was negatively correlated with insulin resistance in high-risk obese women. PrAO-dependent activation of glucose uptake was negatively correlated with body mass index and reflected the decrease of insulin responsiveness of human fat cells with increasing obesity. Methylamine exhibited antilipolytic properties in adipocytes but was unable to directly activate insulin secretion in isolated pancreatic islets. **Conclusions:** PrAO activation by its substrates, e.g., methylamine, increases glucose utilization in human adipocytes in a manner that is linked to insulin responsiveness. Methylamine/PrAO interaction can therefore contribute to adipose tissue enlargement but should be considered as potentially useful for diabetes prevention since it could limit lipotoxicity and facilitate glucose handling, at the expense of favoring healthy fat accumulation.

## 1. Introduction

Having the chemical formula of CH_3_NH_2_, methylamine is the simplest methylated amine that belongs to the class of organic osmolytes, which are low-molecular weight compounds influencing the properties of biological fluids and maintaining cell integrity. In fact, methylamine is a volatile compound present in nature either as a gas or soluble in blood, sweat, urine, and breath. Alongside with trimethylamine and trimethylamine oxide (TMAO), methylamine can give a fishy odor to these liquids. It is currently accepted that, when TMAO blood levels are elevated, there is an increased risk of cardiovascular events [1]. This may be the case after the consumption of foods containing carnitine or lecithins, and their subsequent conversion by human gut microbiome, since TMAO is purported to alter cholesterol metabolism [2]. However, in the case of methylamine, much less is known. In man, it is only admitted that its urinary excretion can be increased after ingestion of creatinine [3,4]. Otherwise, several microorganisms can grow when using methylamine as the sole source of carbon and nitrogen [5,6,7,8] while many other bacterial strains produce methylamine as one of the multiple volatile organic compounds they release in their environment. In several cases, such production is endowed with antimicrobial activity [9], controlling thereby the biodiversity of the surrounding bacterial population (either in the gut or in foods during their processing). Methylamine content of coffee, bacon, bread, and fruits is averaging 51, 24, 12, and 2.5 mg/kg, respectively [10] and is even higher in kola nuts [11]. Present in food, methylamine is also an endogenous amine in pluricellular organisms, including humans. In man, the mean daily excretion of methylamine is 11 mg, and although highly variable, this excretion is of endogenous origin mainly with only subtle contributions from the diet [3].

In biochemical research, high doses (10 mmol/L) of methylamine are routinely used to inhibit lysosomal activity by dissipation of the pH gradient across acidic cellular compartments [12]. However, more physiologically relevant is the observation of the group of L. Raimondi, indicating that methylamine might participate in controlling food intake in rodents [13,14]. This finding has been extended later to birds [15] but is not extrapolated to humans so far. Suspected together with nitrite salts to be implied in deleterious nitrosation triggering gastric tumor formation [16], methylamine is currently supposed to interact with the gut microbiome. However, methylamine is also known from a long while to be oxidized by amine oxidases [17] and to interact with trace amine receptors and aminergic transporters. Its related molecules such as trimethylamine exhibit dramatic changes in their metabolism under diabetic conditions [18]. Such interplay with alterations of osmoregulation and glucose handling in diabetes, together with our previous observations showing that in vivo administration of methylamine reduces the hyperglycemic excursion during a glucose tolerance test in rabbits [19], encouraged us to further study the putative links between methylamine, its oxidation, and insulin sensitivity or secretion.

It is recognized that methylamine is oxidized in mammals by amine oxidases distinct from monoamine oxidases (MAO) since they do not use FAD as a cofactor. In fact, it is copper-containing amine oxidases (AOC) that oxidize methylamine. One of them has been previously called semicarbazide-sensitive amine oxidase (SSAO) since its activity is abolished by semicarbazide. This membrane-bound enzyme, expressed in various anatomical locations, including the vessels at the sites of inflammation is also known as vascular adhesion protein-1 (VAP-1) [20]. SSAO/VAP-1 is a multi-function enzyme with a shed form soluble in plasma [21]. Numerous reports converge to demonstrate that this soluble SSAO/VAP-1 is increased in diabetic conditions [22,23] but the interpretations diverge regarding its role in disease progression. Since the oxidation of its substrates methylamine and aminoacetone produces formaldehyde and methylglyoxal, it has been proposed that sustained inhibition of its activity could limit the levels of these deleterious agents and could reduce the cardiovascular complications of diabetes [24]. However, in experimental models of type 1 diabetes, semicarbazide treatment has never reduced hyperglycemia while tyramine did [25]. More recently, it has been proposed that the serum VAP-1/SSAO concentration is increased in subjects with pre-diabetes in order to counteract hyperglycemia since its increase accompanies that of adiponectin (an adipokine that improves insulin sensitivity) and is negatively associated with incident diabetes [26].

Other recent findings led us to establish a working hypothesis presuming that some of the methylamine actions could be related not with plasma SSAO/VAP-1 but with that present in adipose tissue: (1) Among the insulin-sensitive tissues, regarding glucose utilization, adipose depots abundantly express SSAO/VAP-1 [27], up to a level that is approximately one thousand times higher than in plasma [28]; (2) in adipose cells, the presence of high concentrations of SSAO/VAP-1 substrates promotes several insulin-like effects such as the stimulation of glucose transport, lipogenesis, and adipogenesis [29]; (3) these somewhat “insulin-like” effects have been demonstrated to depend on the hydrogen peroxide generated by amine oxidases irrespective of the oxidized substrate [30].

All these issues justified the verifications we performed in the present work in order to evidence a link between the methylamine actions in adipose tissue and the overall insulin sensitivity. However, before the presentation of our data, mainly collected in humans, it is important to state a nomenclature-related point: Many novel pharmacological agents have been developed during the least decade to inhibit AOCs in a more selective and/or potent manner than semicarbazide [31]. A list of these inhibitors is available in [32]. Consequently, SSAO has been renamed to primary amine oxidase (PrAO) [33], a designation used hereafter, though semicarbazide was still used as a reference inhibitor of its activity in the present study.

The following results will not depict the exact mechanisms of methylamine action since this small natural molecule is able to interact with a myriad of targets. However, they bring preclinical evidence that such naturally occurring PrAO substrate may facilitate glucose handling in normal, pre-diabetic and/or overweight individuals since it activates glucose transport and inhibits lipolysis in adipocytes, while it does not improve insulin secretion by pancreatic islets. Our compiled observations also suggest that adipose AOCs involved in methylamine metabolic actions decrease with increasing insulin resistance while methylamine metabolic effects appear to be impaired with extended fattening.

## 2. Materials and Methods

### 2.1. Chemicals and Reagents 

Methylamine, benzylamine, semicarbazide, cytochalasin B, fatty acid-free bovine serum albumin, hydrogen peroxide, horseradish peroxidase, isoprenaline, Cytochalasin B, semicarbazide, and routinely used chemicals were from Sigma-Aldrich-Merck (St. Quentin Fallavier, France), unless otherwise specified. Amplex Red (10-acetyl-3,7 dihydrophenoxazine from FluoProbes) was provided by InterChim (Montluçon, France). [^3^H]-2-deoxyglucose (2-DG, 26 Ci/mmol) and [^14^C]-benzylamine (NEC 835050UC) were purchased from PerkinElmer Life and Analytical Sciences (Boston, MA, USA). 

### 2.2. Subjects and Preparation of Adipose Samples

Caucasian premenopausal severely obese women, who were candidates for biliopancreatic diversion with duodenal switch surgery [34], provided their written informed consent for the study performed at the Quebec Heart and Lung Institute (Québec, Canada). Subjects with prior weight-loss surgery, a history of hepatitis, smoking, and/or consuming more than 100 g of alcohol per week were excluded. The experimental design was approved by Laval University Ethics Committee (approval # CERUL 2004-108). Blood sampling was performed in the morning after a 12 hour-overnight fast just before surgery. Fasting glucose concentrations were evaluated by routine methods in the clinical laboratories of the Quebec Heart and Lung Institute. Plasma insulin concentrations were determined by a high-sensitivity enzyme-linked immunoabsorbent assay (ELISA; Cedarlane Laboratories Ltd., Hornby, Ontario, Canada). Insulin resistance based on the homeostasis model assessment (HOMA) index was evaluated according to the following equation: Insulin (µUI/mL) × glucose (mmol/L)/22.5 [35]. After surgical excision under general balanced anesthesia (barbiturate/fentanyl/nitrous oxide), subcutaneous adipose tissue (ScAT) samples (approx. 2 g) were obtained from the superficial abdominal fat depot (near the umbilicus), and were frozen in liquid nitrogen and then stored at −80 °C until use. 

For a second set of experiments, samples of subcutaneous adipose tissue (ScAT) were obtained from patients undergoing abdominal lipectomy at the plastic surgical department of the Rangueil Hospital (CHU Toulouse, France) in accordance with the local ethic committee and INSERM guidelines under the agreement reference DC-2008-452. The adipocyte preparations used in the present study were obtained from a total of 56 women of a mean age of 40 years with a mean body mass index (BMI) of 25. The fat cells were immediately subjected to hexose uptake assays as described below since freezing/thawing sequences are not compatible with the preparation of functional adipocytes, as described in [36].

### 2.3. Animals 

The experiments were carried out following the protocols and ethical requirements approved by the Committee for the Care and Use of Animals of the University of Salamanca, in accordance with the regulations for the use of animals in research studies established by the EU Directive (2010/63/EU) and Spanish legislation (RD 53/2013) approved on 1 February 2013. Twelve male New Zealand white rabbits aged 7–8 months (body weight between 2.5–3.0 kg) were euthanized for obtaining functional isolated pancreatic islets and adipocytes. A total of twenty C57BL/6 mice of both sexes were used for pancreatic islet preparation and insulin secretion measurements. All animals were maintained under controlled conditions, at 21 ± 2 °C, with 50% ± 5% relative humidity, a 12/12 h light–dark cycle and with free access to food (irradiated chow) and water. 

### 2.4. Methylamine Oxidation by Human ScAT

Human ScAT samples were thawed and homogenized at room temperature in 200 mM phosphate buffer (pH 7.5 during 30 s) just before assay of hydrogen peroxide release to avoid the formation of a fat cake that is solid at cold temperatures and hampers enzymatic reactions.

Activity of human PrAO was assessed by determining the hydrogen peroxide released by methylamine oxidation in homogenates of thawed ScAT. A fluorimetric method originally reported for MAO activity assay [37], was slightly adapted to PrAO as already described [38]. Hydrogen peroxide release was detected with a chromogenic mixture (4 U/mL horseradish peroxidase plus 40 μM of the fluorescent probe Amplex Red) in phosphate buffer, pH 7.5 and quantified with hydrogen peroxide standard curve (from 0.05 to 5 µM concentrations). Homogenates were distributed in 96-well dark microplates (at approximately 100 µg protein/well) and incubated at 37 °C in 200 µL final volume after pre-incubation without (control) or with 1 mM semicarbazide (abolishing the SSAO activity). Fluorescence data (ex/em: 540/590 nm) were collected on a Fluoroskan Ascent microplate reader (ThermoLabsystems, Finland). Increasing concentrations from 0.05 to 2 mM methylamine indicated that maximal velocity was reached at 1 mM. PrAO activity was therefore determined at this concentration in the cohort study.

### 2.5. Adipocyte Preparation, Glucose Transport, and Lipolysis Assays

Human adipocytes were isolated from ScAT as previously described [39] after digestion by 15 µg/mL type TM liberase (Roche Diagnostics, Meylan, France) at 37 °C in Krebs–Ringer buffer with 15 mM bicarbonate, 10 mM HEPES, and 3.5% of bovine serum albumin (KRBH buffer, pH 7.4). Filtration through pieces of nylon stockings and two washes of the buoyant fat cells allowed us to obtain preparations of human adipocytes that were highly responsive to insulin, regarding stimulation of hexose transport, assessed by a radiometric method based on the uptake of the non-metabolizable [^3^H]-2-deoxyglucose (2-DG). Briefly, a [^3^H]-2-deoxyglucose isotopic dilution was added at a final concentration of 0.1 mM (around 1,000,000 dpm/vial) to 400 µL of cell suspension after 45 min preincubation with the tested agents. Human adipocytes were then incubated for 10 min, and assays were stopped with 100 µL of 100 µM cytochalasin B. Aliquots (200 µL) of cell suspension were immediately centrifuged in microtubes containing dinonyl phthalate of density 0.98 g/mL, thus allowing the separation of buoying adipocytes, which were counted in scintillation vials as previously described [40]. Lipolytic activity was assessed by the determination of glycerol release in KRBH buffer, but in the presence of 6 mM glucose and after 90 min incubation of 400 µL adipocyte suspensions with the indicated concentrations of the tested agents, as depicted in [41]. Similar protocols were followed for the preparation and incubation of rabbit adipose cells with slight modifications, as reported in [42].

### 2.6. Insulin Secretion by Isolated Pancreatic Islets 

Islets were isolated by collagenase digestion of the rabbit or mouse pancreas. After isolation performed as previously reported [43], islets were first pre-incubated for 60 min at 37 °C in a bicarbonate buffer medium, pH 7.4, containing 15 mM glucose and supplemented with 1 mg/mL bovine serum albumin fraction V (Boehringer Mannheim, Germany). As in [44], batches of three islets were then incubated for one hour in 1 mL with 8 mM glucose alone or together with methylamine or with the reference secretagogues carbachol and forskolin. At the end of the incubation, released insulin was determined with a radioimmunoassay kit (CIS Radioquímica-Schering, Spain) as detailed in [45]. 

### 2.7. Oxidation of Radiolabeled Benzylamine by Adipose Tissue Homogenates

Amine oxidase activity was measured on homogenates of thawed adipose tissue with 0.5 mM [^14^C]-benzylamine as labeled substrate in 200 mM phosphate buffer, pH 7.5 for 30 min as previously described [46].

### 2.8. Statistical Analysis

Experimental data are given as mean ± SEM of the indicated number of independent observations and were analyzed with IBM SPSS Statistics, version 25.0 (IBM Corp, Armonk, NY, USA) by a one–way ANOVA or paired Student’s *t*–test when appropriate. Statistical significance was assumed when *p* < 0.05. 

## 3. Results

### 3.1. In Vitro Oxidation of Methylamine by Adipose Tissue Preparations from High-Risk Obese Patients

Although studied in various mouse and rat models of diabetes and obesity, methylamine actions have been poorly investigated in humans. As we have already reported that human adipose depots are able to oxidize methylamine similarly to benzylamine, another natural primary amine [41], it was decided to further explore this metabolism in obese patients with overt insulin resistance. Samples of subcutaneous adipose depots were obtained from twelve severely obese women undergoing bariatric surgery who were hyperinsulinemic (Table 1).

**Figure 1:**
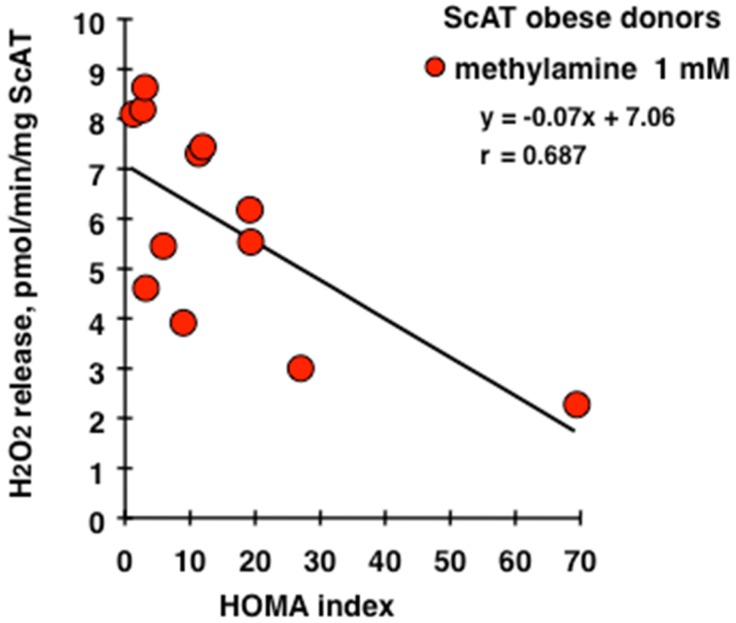
Correlation analysis for methylamine-stimulated hydrogen peroxide release by subcutaneous adipose tissue (ScAT) preparations in function of the HOMA index of insulin resistance. When measured in identical conditions, the oxidation of 1 mM methylamine by homogenates of frozen ScAT released different amounts of hydrogen peroxide depending on the donor. Among the group of twelve obese women depicted in Table 1, these hydrogen peroxide productions were plotted in function of HOMA, an index of insulin resistance. The correlation coefficient *r* of the linear regression indicated a significant negative relationship at *p* < 0.02.

The addition of 1 mM methylamine to thawed ScAT preparations of these patients released hydrogen peroxide in a manner that was significantly sensitive to semicarbazide inhibition: 6.2 ± 0.7 and 1.5 ± 0.2 pmol/mg ScAT/min for methylamine 1 mM and methylamine + semicarbazide 1 mM, respectively (*n* = 6, *p* < 0.001). In contrast, MAO inhibition impaired only marginally the methylamine oxidation: Hydrogen peroxide release in the presence of methylamine + pargyline 1 mM was 5.6 ± 0.5 pmol/mg ScAT/min. The increase in baseline hydrogen peroxide release was detectable only at 0.1 mM methylamine: 1.9 ± 0.2 vs. 2.7 ± 0.4 pmol/mg ScAT/min (*n* = 6, *p* < 0.05). All these characteristics indicated that methylamine oxidation was supported by PrAO. Deeper analysis showed that methylamine-induced hydrogen peroxide release was not more elevated in the individuals with the higher insulin resistance. Surprisingly, there was a negative relationship between the maximal capacity of ScAT to oxidize methylamine and the HOMA of the donor (Figure 1). Less amazing was the lack of correlation between this PrAO activity in ScAT and BMI (not shown, see graphical abstract), which was probably due to the high values of BMI characterizing the patients selected for this pilot study (all >40, belonging to the class of high-risk obesity).

### 3.2. Stimulation by Methylamine of Glucose Transport in Adipose Cells from Non-Obese, Overweight, and Obese Patients

**Figure 2:**
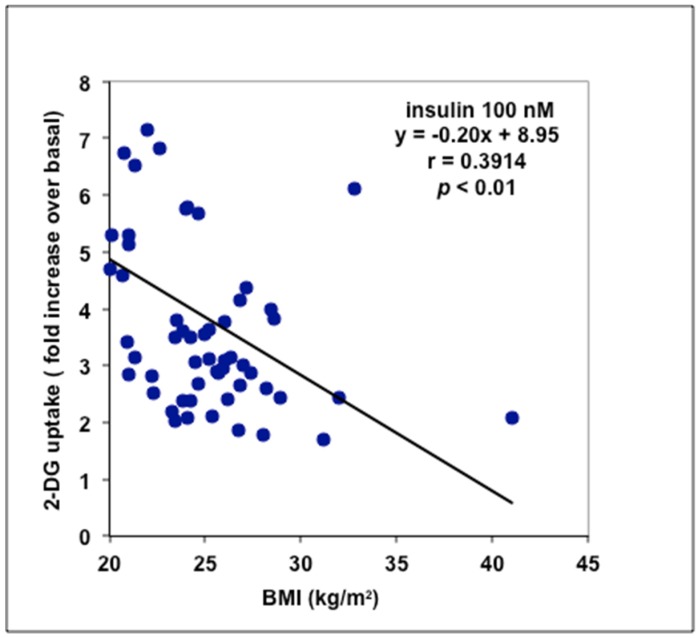
Relationships between obesity (assessed as the body mass index, BMI) and insulin stimulation of glucose transport in adipocytes freshly isolated from abdominal ScAT. Effect of 100 nM insulin is expressed for each individual as fold increase over basal hexose uptake. Linear regression equation and *p* value refer to 56 individuals, characterized by a mean value for baseline 2-DG uptake of 0.55 ± 0.03 nmol/100 mg lipids/10 min.

Since adipose cells could not be prepared immediately upon surgery in the group of high-risk obese patients, further exploration of biological effects of methylamine was performed in another sample of individuals with BMI ranging from 20 to 41 kg/m^2^. A total of 56 patients were included in this study: All were women, aged from 20 to 63 (mean age: 40 ± 1 year, mean BMI: 25.0 ± 0.5). In this second set of experiments, the vicinity between the laboratory and the surgery department allowed a rapid treatment of portions of removed fat samples by liberase digestion: Preparation of functional adipocytes was achieved in less than two hours following surgical interventions. Unfortunately, only an anthropometric index of obesity (BMI) and not biochemical circulating parameters such as fasting blood glucose and insulinemia could be collected from these donors. The insulin-like effect of methylamine on glucose transport into adipocytes was compared to baseline (negative control) and insulin-stimulated uptake (positive control). Although most of the women undergoing plastic surgery were classified as normal weight or overweight, there were four individuals with low-risk obesity, with BMI comprised between 30 and 41. Insulin stimulation of hexose uptake behaved as a response exhibiting high inter-individual variation and showing a significant negative relationship with BMI at *p* < 0.001 (Figure 2). High responders (i.e., those whose insulin-dependent activation of glucose transport was higher than a fourfold increase over baseline) were essentially found in the class of normal weight, while three of the four obese patients showed lower insulin responsiveness (limited to a doubling of basal 2-DG uptake). The subcutaneous adipocytes of overweighed women exhibited intermediate insulin responsiveness, hardly reaching a fourfold activation of basal hexose uptake. 

**Figure 3:**
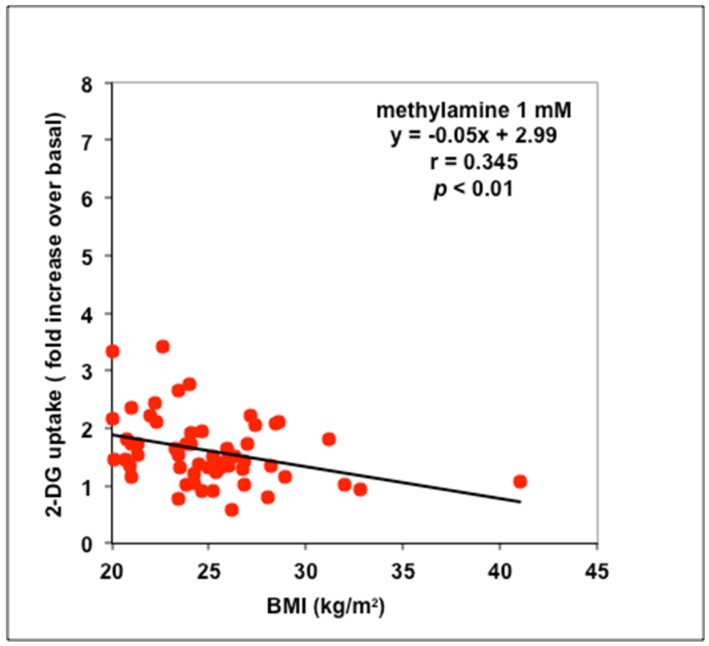
Relationships between BMI and methylamine stimulation of glucose transport in adipocytes isolated from abdominal ScAT. Linear regression equation and *p* value refer to the same 56 individuals plotted in Figure 2.

The insulin-like effect of methylamine was examined in these conditions and also exhibited significant negative relationship with BMI (Figure 3). In contrast to insulin, many adipocyte preparations did not activate glucose transport in response to methylamine. Such low responders to methylamine were found in each class of BMI (20–25, 25–30, and >30). As with insulin, the high responders were essentially subjects with normal body weight.

**Figure 4:**
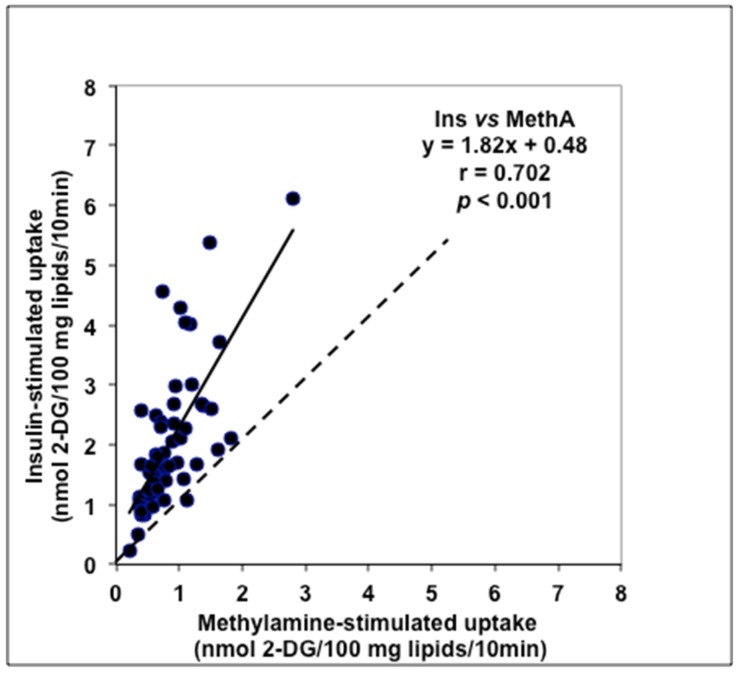
Relationships between insulin (Ins) and methylamine (MethA) stimulation of glucose transport in adipocytes isolated from abdominal ScAT. Dotted line represents identity line for the two variables. Linear regression equation (black line) and *p* value refer to 56 individuals, showing mean ± SEM values of 2-DG uptake of 2.03 ± 0.16 and 0.85 ± 0.06 nmol/100 mg lipids/10min for 100 nM insulin and 1 mM methylamine, respectively.

Plotting the insulin responses versus the methylamine responses of adipocytes from this cohort clearly indicated that a positive linear regression gives a significant estimation of the relationship detected between these two variables (Figure 4). Although the regression line was not superimposed with the identity line (most of the adipocyte preparations were much more responsive to the pancreatic hormone than to the primary amine), the methylamine effect on glucose transport in human fat cells can be qualified as insulin-like or partially insulin-mimicking. 

The adipocytes that did not respond to insulin did not respond to methylamine. Since the activation of glucose uptake by methylamine and benzylamine has been reported in rodent adipocytes to be dependent on PrAO oxidation, generation of hydrogen peroxide, vanadium oxidation, and the subsequent recruitment of the insulin-regulated glucose transporters GLUT4 at the cell surface [47], it can be supposed that a similar mechanism is implied in human fat cells. Thus, the individuals exhibiting the higher responses to both insulin and methylamine were probably those with the lower insulin resistance and thereby with the lower HOMA index. This interpretation therefore totally agrees with that raised from the analysis of the relationship between PrAO activity and HOMA index in the group of high-risk obese women.

### 3.3. Comparative Examinations of Methylamine Actions in Human Adipocytes

**Figure 5:**
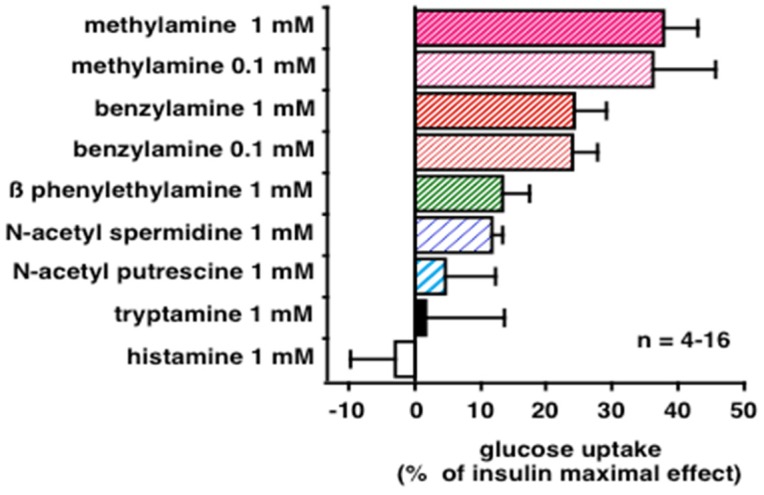
Effect of natural amines on glucose uptake in human adipocytes. Suspensions of human fat cells (15–20 mg lipids/400 µL) were incubated 45 min without (basal, 0%), with 100 nM insulin (max, 100%) or with the indicated concentrations of amines, then [^3^H]-2-deoxyglucose uptake was assayed on 10-min period. Mean ± SEM of 4–16 adipocyte preparations.

Since the methylamine is too small/simple of a molecule (CH_3_NH_2_) to successfully generate a pharmacophore for screening and optimization of more complex compounds sharing PrAO substrate properties and insulin-like effects, it was of interest to check whether other natural amines could reproduce the effects of methylamine on glucose uptake in human adipocytes. We limited such comparison to representative members of the families of aromatic amines, polyamines, and aminergic modulators, by testing them in several of the 56 subjects of the above reported cohort, when the available time and biological material were sufficient to deeper explore adipocyte functionalities. Figure 5 shows that among the natural amines tested, methylamine and benzylamine were unambiguously the most potent in activating hexose uptake in freshly isolated human adipocytes. Of note, both of them are reference substrates for human PrAO (identical to SSAO/VAP-1, encoded by the *AOC3* gene). β-phenylethylamine, which is present in chocolate or fermented cheeses, was also able to activate glucose uptake. Also known as phenetylamine, this precursor of psychoactive drugs can be metabolized by MAO and PrAO. Polyamines and tryptamine were less efficient. Intriguingly, histamine, which is a recognized substrate of a copper-containing amine oxidase distinct from PrAO, namely diamine oxidase, product of AOC1 gene, did not clearly activate glucose uptake in these conditions (Figure 5).

Other complementary investigations of the insulin-mimicking actions of methylamine were performed. First, there was not any significant effect of methylamine on glucose uptake when present at micromolar doses, an indication in agreement with the recognized Km values for methylamine for PrAO, which are in the 100–800 µM range [17]. Then, the effect of a millimolar dose of methylamine on hexose uptake was significantly inhibited when its oxidation was abolished by semicarbazide plus pargyline, resulting in a decrease from 26.1% ± 4.3% to 5.9% ± 3.9% of insulin maximal activation of glucose transport (*n* = 8, *p* < 0.01). Taken together, all these verifications confirm that the activation of hexose uptake by methylamine depends on its oxidative deamination, and agree with the already reported disappearance of methylamine insulin-like effects in AOC3 knock-out mice [48,49].

Moreover, Table 2 shows that, when glycerol release into the incubation medium of isolated adipocytes was used as an index of lipolytic activity, the β-adrenergic agonist isoprenaline was dose-dependently stimulatory. Submaximal stimulation of lipolysis by 100 nM isoprenaline was impaired by 1 mM methylamine or benzylamine to a similar extend, while brimonidine, a potent and selective α_2_-adrenergic agonist was more antilipolytic, even at 1 µM. As expected, the β-adrenergic activation of lipolysis by isoprenaline was totally blunted by the selective β-adrenergic antagonist, bupranolol (Table 2). This comparative approach indicated that another biological response of adipocytes triggered by methylamine consists in lipolysis inhibition, which, alongside the triacylglycerol synthesis, is a well-recognized insulin anabolic action.

### 3.4. Lack of Methylamine-Induced Insulin Secretion in Pancreatic Islets

Adipose tissue contributes to the control of glucose homeostasis, as it is one of the targets of insulin-dependent activation of glucose transport, together with heart and muscles. However, there are many other ways to facilitate insulin hypoglycemic action, such as optimizing its secretion by pancreatic islets (e.g., incretin effect). It was therefore tested whether methylamine could mitigate insulin resistance via a direct facilitation of insulin secretion. To avoid in vitro tests using cell lineages of human pancreatic β-cells, which sometimes work differently from native endocrine cells, and often lose SSAO/VAP-1 expression in culture conditions [50], it was decided to work with freshly isolated rabbit islets, in view of the improvement of glucose utilization we previously demonstrated in vivo for methylamine in this animal model [19].

**Figure 6:**
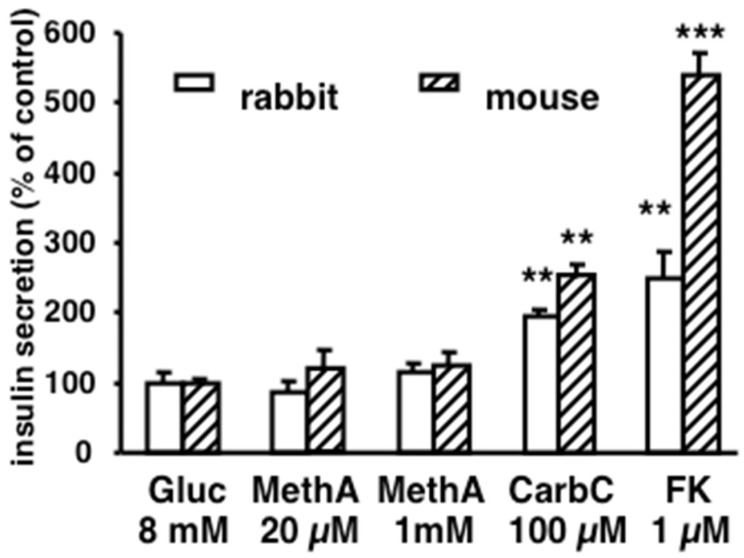
Absence of methylamine direct activation of insulin secretion by islets of Langerhans isolated from rabbit (open bars) or mouse pancreas (cross-hatched bars). Values represent mean ± SEM of insulin release by islets incubated for 1 h with 8 mM glucose (Gluc) without (control set at 100%) or with the indicated doses of methylamine (MethA), carbachol (CarbC), or forskolin (FK). For each condition, 15–23 batches of islets were used. Basal insulin release was 16.5 ± 1.2 µIU/mL/islet/h for rabbit and 13.9 ± 1.6 µIU/mL/islet/h for mouse. Significantly different from control at: ** *p* < 0.01; *** *p* < 0.001, by *t* test.

In isolated rabbit islets that were responsive to forskolin and carbachol regarding insulin secretion stimulation, there was no influence of methylamine, either at 20 µM or at 1 mM (Figure 6). Thus, a direct activation by methylamine of pancreatic PrAO or of any other target involved in insulin secretion pathway apparently did not contribute to the improvement of glucose handling by methylamine. 

However, before discarding a putative direct action of methylamine on insulin secretion, it was verified whether a similar lack of “incretin-like” occurred in another animal model. A similar situation was observed in mouse islets: The two secreting agents of reference (CarbC, FK) enhanced insulin secretion while methylamine was without influence (Figure 6).

### 3.5. Methylamine is Interacting with Rabbit Amine Oxidases and Adipocytes 

**Figure 7:**
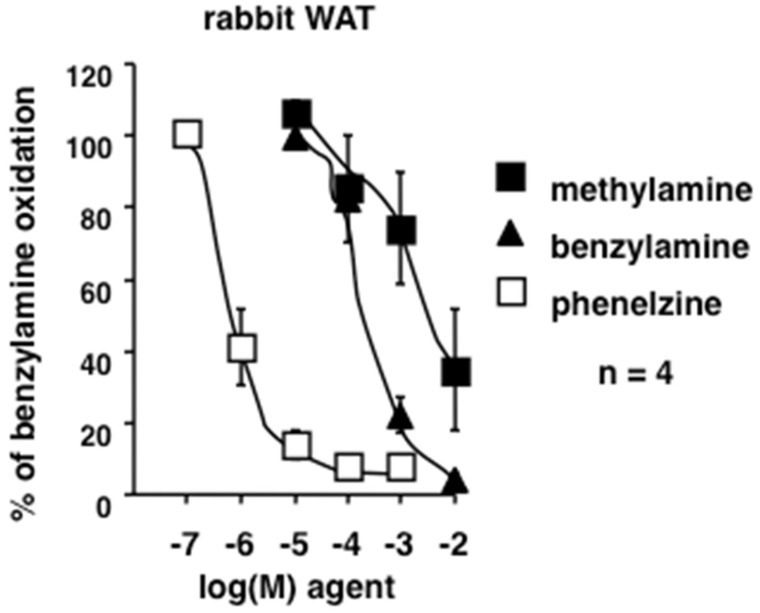
Competition by phenelzine and natural amines of the [^14^C]-benzylamine oxidation by rabbit white adipose tissue (WAT) preparations. Rabbit WAT homogenates were incubated for 30 min in the presence of 0.5 mM [^14^C]-benzylamine without (100%) or with increasing concentrations of phenelzine (open squares), benzylamine (black triangles), or methylamine (black squares). Mean ± SEM of four observations.

Several verifications were performed in rabbit white adipose tissue (WAT). It was tested whether methylamine was a substrate of rabbit PrAO by competition experiments. The in vitro oxidation of [^14^C]-benzylamine was inhibited by low doses of phenelzine, a non-selective irreversible inhibitor of amine oxidase (Figure 7). Cold benzylamine dose-dependently competed for radiolabeled benzylamine oxidation, as it was also the case for methylamine (Figure 7). At 10 mM, methylamine impaired most of the benzylamine oxidation, likely resulting from a competition for the PrAO catalytic site. This indicated that, as already reported for other species, methylamine is readily oxidized in rabbit WAT. 

Then, it was searched whether methylamine could improve glucose uptake in rabbit adipocytes. The baseline uptake of non-metabolizable hexose was 0.33 ± 0.07 nmol 2-DG/100 mg lipids/10 min, it was increased up to 0.70 ± 0.20 by methylamine 1 mM, and reached 3.24 ± 0.63 nmol 2-DG/100 mg lipids/10 min with 100 nM insulin (*n* = 4, *p* < 0.01). Lastly, it was observed that 1 mM methylamine hampered the stimulatory effect of the lipolytic agent forskolin, since only 74.0 ± 4.4% of the glycerol released by the diterpene was found in the presence of the natural amine (*n* = 7, *p* < 0.001). Thus, methylamine, which increases glucose tolerance in rabbits, [19], was unable to enhance insulin secretion by pancreatic islets in vitro, while it moderately mimicked insulin action in adipocytes, by partially stimulating glucose uptake and inhibiting lipolysis.

It was therefore observed for the first time in the same animal species that methylamine, a proposed physiological substrate of PrAO, acted more as an “insulin-mimicking” than as an “insulin-secreting” agent, likely as a consequence of its oxidation by adipose amine oxidases and subsequent activation of glucose uptake and inhibition of lipolysis.

## 4. Discussion 

In the present study, the measurement of methylamine oxidation by ScAT preparations from high-risk obese women confirmed that most of the hydrogen peroxide produced in response to the amine is due to PrAO activity since being sensitive to semicarbazide. Moreover, our correlation analyses in obese patients with diabetic complications unexpectedly indicated that the higher was the overall insulin resistance, the lower was the capacity of subcutaneous fat depot to oxidize methylamine. To date, it was supposed that methylamine oxidation was increasing with the evolution of diabetes [24], associated with an enhancement of the effects of its deleterious oxidation products on vasculature [51]. 

On the contrary, our observation suggests a novel link between PrAO activity and the progression of diabetic condition. Methylamine oxidation and subsequent stimulation of glucose utilization is optimal in pre-diabetic and pre-obese states, then it appears to decline with the development of fattening and insulin resistance. This novel insight is close to that recently proposed by H-Y. Li and co-workers, based on a six-year follow up study of 600 subjects, and indicating that the circulating soluble form of SSAO/VAP-1 is increased in pre-diabetic states to counteract hyperglycemia and is associated with incident diabetes negatively [26]. In other words, increased PrAO (either in WAT or in plasma) should be considered as a compensatory mechanism useful to limit/delay the evolution to severe insulin resistance state. By providing substrate to PrAO, methylamine could help insulin in facilitating glucose utilization. Indeed, it has been well demonstrated that in adipocytes, the generation of hydrogen peroxide by amine oxidation is influencing insulin signaling and, in the presence of vanadium, leads to the translocation of glucose transporters to the cell surface, thereby reproducing part of the stimulation triggered by the pancreatic hormone [47]. Once overpassed when disease progression is accelerated by other factors (over-nutrition, lifestyle, ageing…), this defense mechanism regresses, accompanied by a decreased PrAO activity, and enhanced insulin resistance. In this view, our analysis reveals that a good responsiveness of adipocytes to insulin is a prerequisite to observe methylamine-dependent activation of glucose utilization.

A concern raised from our correlation analyses is that there was no evident link between the PrAO activity in ScAT and BMI in the group of high-risk obese patients. However it must be noted that BMI ranged between 40 and 80 in this group, suggesting that the subcutaneous adipose depot mass was already beyond a threshold, for which no concomitant increase of fat mass and decrease of insulin sensitivity could be as evident as during the onset of obesity, between overweight and low-risk obese states. At least, the PrAO-dependent insulin-like effect of methylamine is so decreased when BMI increases from 20 to 30 that it can be hardly expected that a further decrease occurs in the upper classes of BMI (from 40 to 80). Another limitation of our study is the lack of complete survey of adipose PrAO expression/activity and of methylamine-dependent activation of hexose uptake in function of a wide range of BMI (from 20 to >40). This defect deserves to be solved by the study of a larger cohort, similar to the survey previously performed on soluble SSAO/VAP-1, and in which a negative relationship could be evidenced between plasma amine oxidase activity and BMI only in women [20,52].

Meanwhile, it can be noted that not any patient of the second cohort studied in Toulouse exhibited a total insulin resistance, since all adipocyte preparations responded to the pancreatic hormone: The lowest detectable activations represented approximately twice the baseline glucose uptake. It will be of paramount importance to determine whether this can be extended to the case of large adipocytes prepared from severely obese, insulin-resistant, patients, as those recruited in Québec. However, one can easily suppose that very large adipocytes of such high-risk obese patients are highly fragile, subject to cell disruption, and unavailable for functional exploration, as it is the case for fat cells isolated from massively obese rodents [53]. For these reasons, a deep decrease in insulin responsiveness is expected to occur in adipocytes from high-risk obese subjects; extrapolating the data we obtained when comparing insulin and methylamine effects in pre-obese and obese women, it can be envisaged that methylamine activation of glucose uptake will almost disappear too. Consequently, any novel approach aiming at combating diabetes on the basis of amine-activated glucose uptake in peripheral tissues appears to be more successful as a preventive treatment than as a curative one. Moreover, the antilipolytic effects of methylamine, primarily observed to occur in human adipocytes in a semicarbazide-sensitive manner [54], were confirmed in the present study. Considering that methylamine counteracted the stimulatory action of the lipolytic agent isobutylmethylxanthine [54] and that of isoprenaline as well, and since its antilipolytic effects are less potent than those mediated by α_2_-adrenoreceptor activation [42], one can conclude that they are not mediated by Gi-coupled receptors. Such antilipolytic capacity is nevertheless useful to limit the deleterious complications of lipotoxicity that emerge with obesity and further indicate a possible usefulness of methylamine as preventing the approach of metabolic diseases.

Contrasting with its insulin-mimicking action in adipocytes, the lack of direct action of methylamine on insulin secretion by pancreatic islets does not support any insulin-secreting properties of this molecule. Importantly, these tissue-specific differences were observed in the same animal species: The rabbit, definitely reinforcing the idea that methylamine oxidation favors glucose utilization rather than facilitating insulin secretion. However, our in vitro observations made independently on different cell types, cannot exclude that the pharmacokinetics of ingested methylamine generates other actions, e.g., in the intestinal tract and its microbiota as well as in other host tissues once adsorbed. In this view, the methylamine derivative TMAO is mainly generated by gut microbiome during the digestion process rather than brought by foods [1], and only minor portions of ingested TMAO are lost in feces, indicating an intense metabolism with fast turnover in the circulation [55]. Thus, the fate of methylamine and its related metabolites sustains how the gut microbiome generates biologically active compounds that circulate in the blood stream and act on diverse organs. For the particular case of TMAO, a positive association between elevated TMAO levels and cardiovascular diseases and complications is now widely accepted [1] and current therapies aim at preventing the appearance of this metabolite in the intestinal tract. In the case of methylamine, our present interpretations do not focus interest on an interplay with the gut microbiome, but prompt us to propose that any increase of the occurrence of this amine around the adipocytes, obtained either by ingestion or endogenous generation, may facilitate fuel utilization and consequently may delay or prevent the progression of cardiometabolic diseases in consumers. As a consequence, the insulin-like effect of methylamine, highly variable in humans, warrants future investigations the putative effects of PrAO-interacting agents and cardiometabolic diseases. Unfortunately, the methylamine molecule is too small/simple to generate a successful pharmacophore for selective targets. Further screening and optimization of compounds selectively sharing the PrAO/SSAO substrate properties and insulin-like effects of methylamine is warranted. It is of interest to check whether other natural amines could reproduce and even overpass the effects of methylamine on glucose uptake in human adipocytes, probably via PrAO activation. Our preliminary test indicated that the domain is open for aliphatic or aromatic amines but also focused attention of the other possible targets of such amines (histamine receptors, adrenoreceptors, trace amine associated receptors…) that may counteract their efficiency in activating glucose uptake in human fat cells, supposed to depend on hydrogen peroxide-induced inhibition of tyrosine phosphatases involved in insulin signaling turn-off [47]. Indeed, hydrogen peroxide, a product of methylamine oxidation alongside formaldehyde, has to be considered as instrumental in the effects of natural amines, endowed so far with only deleterious consequences.

## 5. Conclusions

In spite of its hypophagic effects in rodents, methylamine has never been tested as an anti-obesity agent in humans, as far as we know. Similarly, in spite of its insulin-like effect described in rodent adipocytes, this primary amine has not been further studied for its potential antidiabetic properties. In this work, we confirm that methylamine is oxidized by PrAO, activates glucose uptake and inhibits lipolysis in rabbit and human adipocytes. However these effects appear to decrease with the degree of obesity or its endocrino-metabolic complications. The stimulatory action of methylamine on glucose transport is insulin-mimicking in nature, as it is highly correlated with insulin sensitivity at the cellular level, while its capacity to be oxidized is negatively correlated with insulin resistance in women. Though methylamine is a small molecule, its interaction with amine oxidases might be subjected to a screening strategy aiming at identifying lead compounds useful to increase/mimic efficacy of conventional therapeutics of diabetic conditions or to reduce their adverse effects.

In all, the above-mentioned relationships between PrAO-dependent methylamine oxidation, adipocyte metabolism, and glucose or lipid handling deserve to be further studied in pathologies such as diabetes or obesity At the present time, inhibition of PrAO activity, either of a pathological or pharmacological nature, should be associated with a limitation of the somewhat insulin-like actions of methylamine and does not appear as a so attractive way of treating metabolic diseases as recently proposed [32].

## Figures and Tables

**Table 1:** Clinical parameters of the group of twelve severely obese patients.

Parameter	Mean Values
Age (y)	36 ± 2 (28–46)
Body weight (kg)	154.0 ± 9.3
Body mass index (kg/m^2^)	58.67 ± 3.24
Fasting glucose (mmol/L)	6.65 ± 1.01
Fasting insulin (µU/mL)	43.56 ± 8.55
HOMA (insulin resistance)	15.3 ± 5.4

Values are means ± SEM of 12 individuals. For age, min and max values are given in parentheses. HOMA: Homeostasis model assessment of insulin resistance.

**Table 2:** Inhibitory effect of methylamine on isoprenaline-stimulated lipolysis in human adipocytes.

Condition	Glycerol Release µmoles/100 mg lipids/90 min	*p* <
Baseline	0.10 ± 0.01	0.000001
Isoprenaline 10 nM (Iso, β-agonist)	0.40 ± 0.08	0.02
Isoprenaline 100 nM	0.67 ± 0.09	Control
Isoprenaline 1 µM	0.73 ± 0.09	NS
Iso 100 nM + benzylamine 1 mM	0.36 ± 0.05	0.003
Iso 100 nM + methylamine 1 mM	0.38 ± 0.06	0.009
Iso 100 nM + brimonidine 1 µM (α_2_-agonist)	0.27 ± 0.05	0.0004
Iso 100 nM + bupranolol 10 µM (β-antagonist)	0.09 ± 0.02	0.00009

Each value is the mean ± SEM of 15 adipocyte preparations containing 18 ± 1 mg of cell lipids per 400 µL assay. Different from positive control, isoprenaline 100 nM (Iso) at the indicated *p* value. NS: Non significantly different.
